# Surgical Performance Is Not Negatively Impacted by Wearing a Commercial Full-Face Mask with Ad Hoc 3D-Printed Filter Connection as a Substitute for Personal Protective Equipment during the COVID-19 Pandemic: A Randomized Controlled Cross-Over Trial

**DOI:** 10.3390/jcm10030550

**Published:** 2021-02-02

**Authors:** Eleni Amelia Felinska, Zi-Wei Chen, Thomas Ewald Fuchs, Benjamin Otto, Hannes Götz Kenngott, Karl-Friedrich Kowalewski, Beat Peter Müller-Stich, Felix Nickel

**Affiliations:** 1 Department of General, Visceral and Transplant Surgery, Heidelberg University Hospital, 69120 Heidelberg, Germany; EleniAmelia.Felinska@med.uni-heidelberg.de (E.A.F.); z.chen@stud.uni-heidelberg.de (Z.-W.C.); thomas.fuchs@stud.uni-heidelberg.de (T.E.F.); b.otto@stud.uni-heidelberg.de (B.O.); Hannes.Kenngott@med.uni-heidelberg.de (H.G.K.); beat.mueller@med.uni-heidelberg.de (B.P.M.-S.); 2 Department of Urology and Urological Surgery, University Medical Center Mannheim, Heidelberg University, 68167 Mannheim, Germany; karl-friedrich.kowalewski@umm.de

**Keywords:** COVID-19, sars-cov-2, laparoscopy, surgical performance, 3D printing, skill assessment, snorkel mask

## Abstract

(1) Background: During the COVID-19 pandemic, shortages in the supply of personal protective equipment (PPE) have become apparent. The idea of using commonly available full-face diving (FFD) masks as a temporary solution was quickly spread across social media. However, it was unknown whether an FFD mask would considerably impair complex surgical tasks. Thus, we aimed to assess laparoscopic surgical performance while wearing an FFD mask as PPE. (2) Methods: In a randomized-controlled cross-over trial, 40 laparoscopically naive medical students performed laparoscopic procedures while wearing an FFD mask with ad hoc 3D-printed connections to heat and moisture exchange (HME) filters vs. wearing a common surgical face mask. The performance was evaluated using global and specific Objective Structured Assessment of Technical Skills (OSATS) checklists for suturing and cholecystectomy. (3) Results: For the laparoscopic cholecystectomy, both global OSATS scores and specific OSATS scores for the quality of procedure were similar (Group 1: 25 ± 4.3 and 45.7 ± 12.9, *p* = 0.485, vs. Group 2: 24.1 ± 3.7 and 43.3 ± 7.6, *p* = 0.485). For the laparoscopic suturing task, the FFD mask group needed similar times to the surgical mask group (3009 ± 1694 s vs. 2443 ± 949 s; *p* = 0.200). Some participants reported impaired verbal communication while wearing the FFD mask, as it muffled the sound of speech, as well as discomfort in breathing. (4) Conclusions: FFD masks do not affect the quality of laparoscopic surgical performance, despite being uncomfortable, and may therefore be used as a substitute for conventional PPE in times of shortage—i.e., the global COVID-19 pandemic.

## 1. Introduction

**Figure 1:**
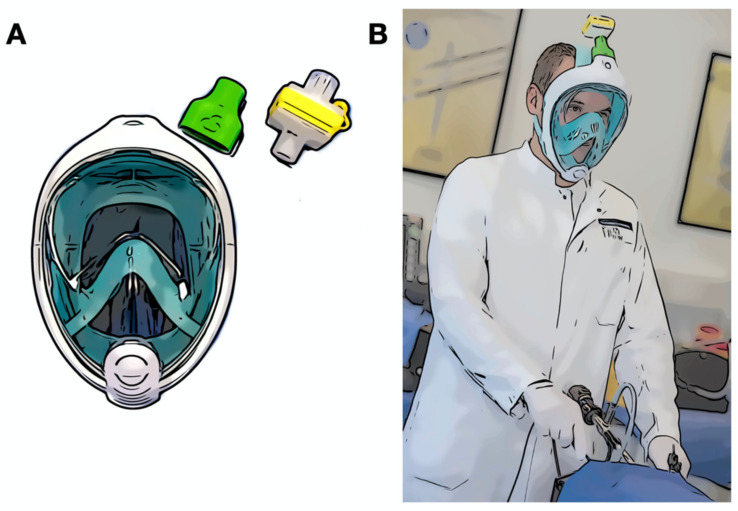
Schematic representation. (**A**) A full-face diving mask with a 3D-printed adapter (green) and air filter (yellow); (**B**) A participant.

As the COVID-19 pandemic has spread, various delivery bottlenecks have become apparent. Not only has there been a shortage of everyday objects, but also shortages of many medical products, especially in the field of respiratory and personal protective equipment (PPE), such as filtering facepiece (FFP) masks. In response to that, inventive minds soon found a promising alternative—full-face diving (FFD) masks, which are suitable for everyday clinical practice after minor adjustments [1,2,3,4,5,6]. Using an ad hoc 3D-printed adapter, a common heat and moisture exchange (HME) respiratory filter could be installed on top of the mask to filter pathogens and allow a clean airflow (Figure 1). Ad hoc 3D printing has enabled the rapid prototyping of solutions in different situations in surgical research with a quick production, highly adaptive modelling, and independence from industrial partners [7]. This has become a popular subject during the COVID-19 pandemic, due to the shortages of PPE and other material in hospitals.

FFD masks have been proposed as a solution for both shortages of conventional non-invasive ventilation material and also the lack of PPE for health care workers. The FFD masks can be reused after a thorough but easy disinfection with a combination of disinfectant wipes for flat surfaces and a liquid disinfectant agent for the breathing channels. The idea quickly became popular through (social) media and was rapidly spread around the world. The principal is particularly appealing to the medical community, especially surgeons and other health care workers in the operative and interventional fields, who work in close proximity to patients’ bodies and are directly exposed to bodily fluids and aerosols. The practicality of such an approach tempted us to dive into clinical implementation. The idea, which may first sound like a joke, has suddenly become a reality—extreme circumstances require extraordinary measures. However, it is not clear if wearing an FFD mask may alter performance during everyday work and therefore compromise patient safety, especially while performing complex surgical tasks. Factors such as the limited view through the visor, the heat, and the restricted air circulation within the mask might greatly affect surgeon’s wellbeing and lead to concentration issues and low performance. To our knowledge, the impact of the use of an FFD mask on the quality of surgical performance has not been evaluated yet. We therefore aimed to assess laparoscopic surgical performance while wearing an FFD mask in a dedicated randomized controlled trial in standardized settings with a homogenous group of participants.

## 2. Experimental Section

### 2.1. Study Design and Tasks

This was a randomized controlled cross-over trial recruiting 40 laparoscopically naive medical students with no known health issues or history of respiratory illnesses. Each participant participated in the study on a voluntary basis and signed an informed consent form. All the participants were recruited from the medical faculty of the University of Heidelberg as part of a clinical elective course over a two-month period. The study took place in the Training Center for Minimally Invasive Surgery (MIC) in the Department for General, Visceral, and Transplant Surgery at Heidelberg University Hospital, Germany. The study was approved by the local ethics committee at Heidelberg University (S-436/2018). After completing a basic laparoscopic training based on the Fundamentals of Laparoscopic Surgery Manual (FLS), the participants were randomized into two groups (with an allocation ratio of 1:1): the FFD mask group and the standard surgical mask group. As PPE, we used the Easybreath© full-face diving mask (Subea^TM^, Decathlon, France). The breathing valve of the mask was fitted with a 3D-printed adapter made out of polylactide (PLA), to the top of which a conventional breathing air filter (HME-Filter) was connected (Figure 1). The adapter was printed according to the freely accessible stereolithography (stl-) files provided by the engineering company Custom Surgical^®^ [8]. The masks were disinfected and prepared according to in-house protocol.

**Figure 2:**
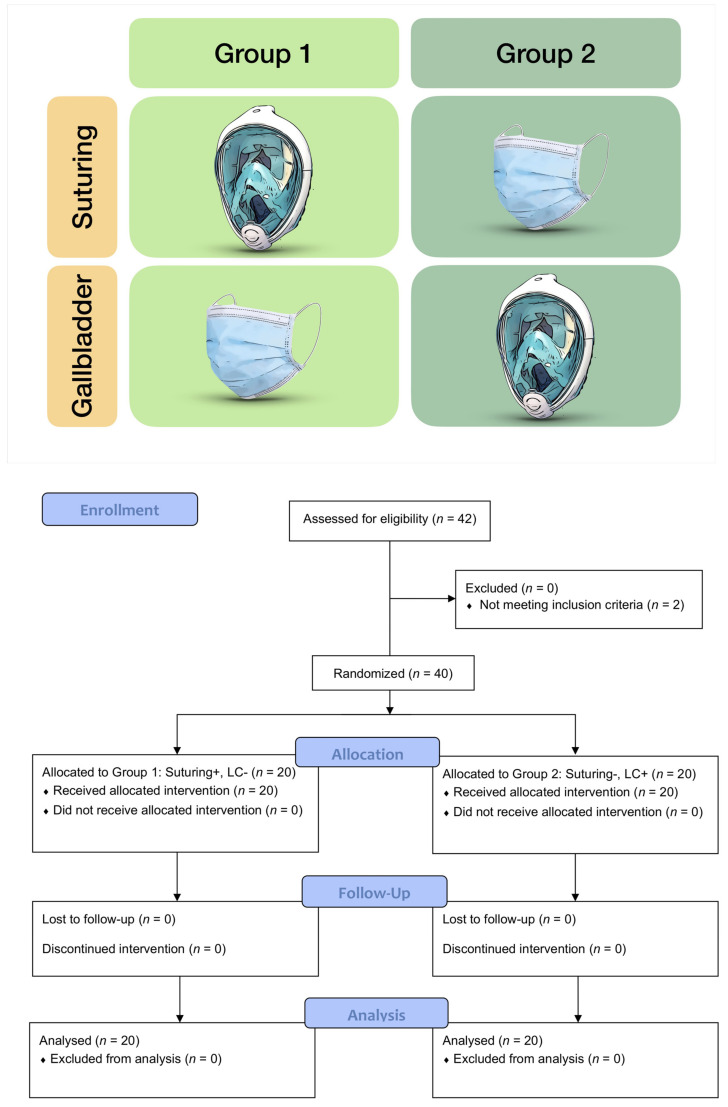
Study design and CONSORT 2010 flow diagram. Group 1 performed the suturing task while wearing the full-face diving mask (+), whereas Group 2 was wearing the surgical mask (−). For cholecystectomy, the masks were swapped.

The students underwent laparoscopic suturing training on standard surgical suture pads until predefined proficiency levels were reached [9,10] and then were asked to perform a laparoscopic cholecystectomy on cadaveric porcine gall bladders [11,12,13]. As the method of instruction, we followed Peyton’s Four-Step Approach (demonstration, deconstruction, comprehension, and execution), as described elsewhere [14,15]. Depending on the assigned group, the participants would only wear the FFD mask for either the cholecystectomy or the suturing task (Figure 2). Global and specific Objective Structures Assessments of Technical Skills (OSATS) checklists were used for the evaluation of suturing and cholecystectomy. The OSATS is a validated assessment tool for rating a student’s skills in laparoscopic cholecystectomies [15]. The global subscale deals with general performance during surgical procedures, such as the handling of instruments and respect for tissue. The specific subscale assesses specific aspects of a laparoscopic cholecystectomy, such as the preparation of the Calot Triangle or the prepping of the gall bladder. Because of its higher construct validity [16], the performance was directly rated by a trained expert using the respective OSATS criteria. For the knot-tying task, the total time needed to reach proficiency and the number of attempts were collected. In addition, all the participants were asked to answer a questionnaire on their personal experience while wearing the FFD mask, with an emphasis on comfort and subjective impact on performance.

### 2.2. Randomization

Upon the completion of the basic laparoscopic training, all the participants were randomized 1:1 into two groups. The randomization numbers were assigned to the participants according to the order of registration for the study. The randomization was conducted through the Research Randomizer program (http://www.randomizer.org) by an independent employee otherwise unconnected to the project. The results of the randomization were sealed in opaque envelopes labeled with consecutive numbers until they were given to the tutor. The envelopes were only opened directly at the beginning of the study for each participant.

### 2.3. Blinding

There was no double blinding during this study. The performance was rated by a trained expert while the participant was completing the task. The participants were instructed not to exchange experiences and information with other participants until the end of the study.

### 2.4. Sample Size

The sample size determination was based on previous studies with identical endpoints, hypothesizing that the surgical masks would be superior to FFD [10].

### 2.5. Statistical Methods

An independent statistician who was otherwise not involved in the study performed the statistical analysis. All the data were entered into a spreadsheet and the statistical evaluation was carried out with R software, Version 3.6.2 [17]. For group comparisons, the non-parametric Mann–Whitney test was applied for continuous and ordinal data. Binary data were analyzed using the Chi^2^ test. Continuous data are reported as medians and ranges. Binary data are reported as absolute and relative frequencies. A *p*-value of less than 0.05 was considered statistically significant.

### 2.6. Harms

Should a participant have, at any point of the study, experienced physical or mental impairments, the training was to be stopped immediately. For personal safety reasons, affected participants would have been excluded from further participation in the study.

## 3. Results

### 3.1. Study Population

A total of 40 laparoscopically naive medical students (out of 42 students screened) were recruited over a two-month period (March to April 2020). They were randomized into the two groups with an allocation of 1:1 and completed the study in June 2020. Group 1 had a mean age of 23.2 ± 3.1 and Group 2 of 22.5 ± 1.9 years. A total of 18 (45%) male and 22 (55%) female students participated (Table 1). Further, we did not include students (*n* = 2) requiring prescription glasses who were unable to switch to contact lenses, as glasses did not fit underneath the snorkel mask. The recruitment period of two months was from March until April 2020.

### 3.2. Laparoscopic Performance Outcomes

**Figure 3:**
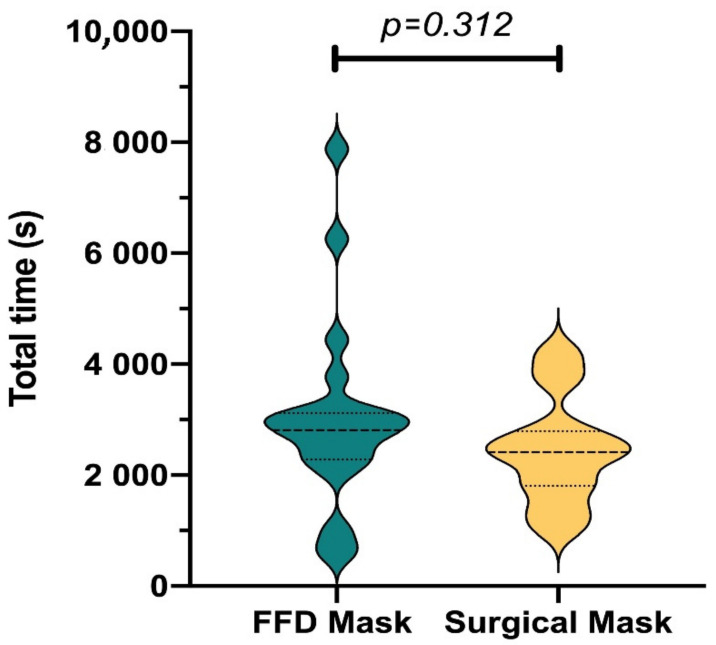
Total time needed to reach proficiency in the laparoscopic knot tying task. s, second.

The suturing task was performed until the participant reached proficiency. Proficiency was defined as making a laparoscopic knot within a two-minute time frame and of a good quality, which was measured with a suturing-specific OSATS checklist [9,10,14]. The mean time until proficiency did not significantly differ between the snorkel mask and surgical mask group (3009 ± 1694 s vs. 2443 ± 949 s; *p* = 0.20). The mean number of attempts until proficiency in suturing was also not significantly different (snorkel mask 12.3 ± 5.45 vs. surgical mask 10.8 ± 3.65; *p* = 0.313). The participants with the highest and lowest number of attempts were featured in the snorkel mask group (*n* = 27 and *n* = 3, respectively). (Figure 3).

**Figure 4:**
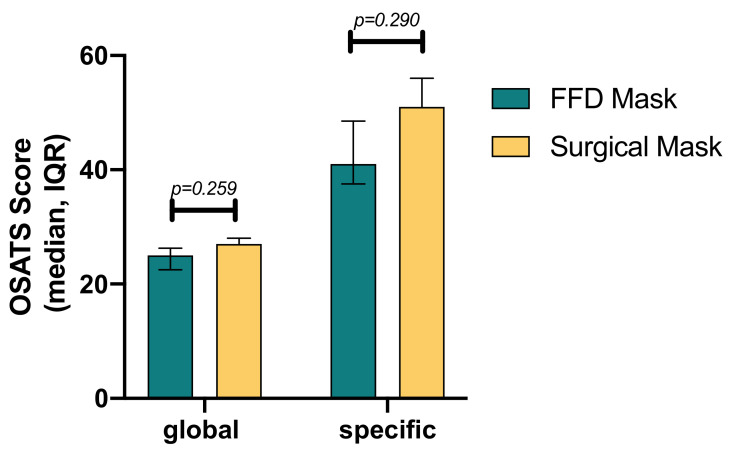
Global and procedure-specific Objective Structured Assessment of Technical Skills (OSATS) score results for laparoscopic cholecystectomy. The median of the OSATS scores and *p*-values are shown. IQR, interquartile range.

For the laparoscopic cholecystectomy task, the medians of the global OSATS scores, with the highest possible score being 35, were not significantly different (snorkel mask 25 (IQR 22.5–26.3) vs. surgical mask 27 (IQR 23.3–28.0); *p* = 0.259). The medians of the task-specific OSATS scores, with the highest possible score being 70, were not significantly different either (41.0 (IQR 37.5–48.5) and 51.0 (IQR 33.5–56.0); *p* = 0.290) (Figure 4). There was no difference in mean time for cholecystectomy between groups (FFD mask 80.3 ± 12.7 min vs. surgical mask 77.8 ± 18; *p* = 0.607).

### 3.3. Full-Face Diving Mask Questionnaire 

**Figure 5:**
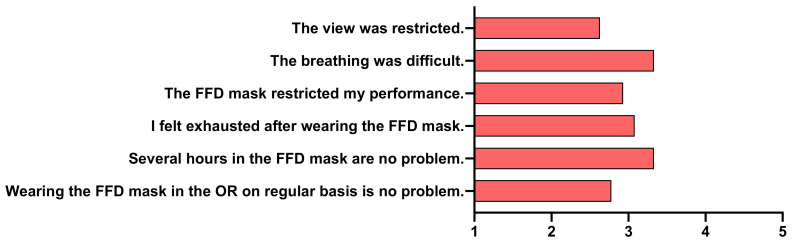
Full-face diving mask questionnaire. Mean Likert score values for each question are shown as bars. FFD, full-face diving.

Concerning the aspect of the comfort of the FFD mask, the participants reported some difficulties in breathing and exhaustion after wearing the FFD mask. However, restriction of view or wearing the FFD mask for longer periods of time were not reported as problems. There was no distinct agreement on whether the FFD mask influenced the participants’ performance. Still, wearing the FFD mask in the OR on a regular basis could have a negative impact according to participants (Figure 5).

## 4. Discussion

The present study did not show any difference between the group wearing an FFD mask with an HME filter and the group wearing a standard surgical mask in terms of the time and number of attempts needed until proficiency in laparoscopic suturing was reached. There was also no significant difference between groups in terms of their mean OSATS scores, completion time, and afflicted damage for the laparoscopic cholecystectomy task. Laparoscopic suturing and laparoscopic cholecystectomy were chosen as representative tasks because intracorporeal suturing is considered an advanced skill indispensable for minimally invasive surgery [18] and cholecystectomies are amongst the most common laparoscopic procedures to be completed in both elective and emergency settings.

Furthermore, the participants did not perceive the FFD mask as a detrimental restriction of performance. However, this observation was restricted only to shorter procedures and the effect of the FFD mask on surgical performance during longer laparoscopic procedures needs to be assessed in further studies. Conversely, Yánez et al. described that wearing conventional PPE, such as FFP2 masks, face shields, and two pairs of surgical gloves, was considered to be uncomfortable and impacted the personal perception of surgical performance negatively [19]. Since the participants in the present study were medical students with little prior experience in the OR, the assessment of the FFD mask could potentially differ when asking practicing surgeons. However, this lack of experience could also suggest that with the frequent use of the FFD mask, its inconveniences could be acclimated to. In some respects, the present study is limited. Since the evaluation of participants’ performance was not blinded and conducted through direct observation, detection bias cannot be completely ruled out. Video-recorded analysis of performance could have been conducted in a blinded fashion, but this was not considered for practical reasons on the one hand. On the other hand, video assessment was not considered because of the superior validity of direct ratings over video ratings, which has been shown in previous studies [16], as well as the inability to assess the amount of help needed, which is an important factor in student trainees.

Further, the environmental influence factors specific to the OR—e.g., machine noises and chatter—did not occur in the training center, which might bias the hearing ability though the mask. Nevertheless, our training center reliably simulates most aspects of the OR, including specific light settings and real instruments. As a limitation of the FFD mask, we found that the voice is muffled by it, making conversations (which are such an indispensable tool during surgery!) harder to understand. Furthermore, surgeons who wear prescription glasses would need to switch to contact lenses. Lastly, the FFD mask did not fully accommodate every face shape despite the three different available sizes. These limitations might require more unconventional solutions. Auditory problems could be mitigated by wearing a small microphone underneath the mask. The improved fitting of the mask could, in critical times, be achieved by using adhesive material. On the other hand, in times of shortage, standard PPE could be reserved to the health care workers not able to use FFD masks.

Presumably, wearing an FFD mask for an extended period of time leads to habituation and diminishes subjective feelings of discomfort. Moreover, the participants stated that they would take into consideration using the FFD mask in the OR even on a regular basis.

In the present study, using an FFD mask as PPE did not significantly affect the quality of laparoscopic surgical performance. The implication of an impairment of patient safety can therefore not be derived from the present study, nor has the equivalence of both masks been definitively proven yet. For this purpose, a larger study population might be considered to test for non-inferiority. Further, this study did not aim to evaluate any of the safety features of the FFD mask, and additional studies concerning the true value of using the FFD mask as PPE during the COVID-19 pandemic need to be conducted.

## 5. Conclusions

Full-face diving masks with ad hoc 3D-printed connections to HME filters do not seem to affect the quality of laparoscopic surgical performance, despite being uncomfortable, and may therefore be used as a substitute for conventional PPE in times of shortage—i.e., during the global COVID-19 pandemic—after confirming its protective features in further studies. This easy-to-assemble PPE might offer a solution to the ethical dilemma of PPE rationing during the COVID-19 pandemic.

## Figures and Tables

**Table 1:** Participants’ general characteristics.

	Total	Group 1	Group 2
*n*, (%)	40 (100)	20 (50)	20 (50)
Age			
Mean (SD)		23.2 (3.1)	22.5 (1.9)
Gender			
Male, *n* (%)	18 (45)	9 (45)	9 (45)
Female, *n* (%)	22 (55)	11 (55)	11 (55)
Other, *n* (%)	0 (0)	0 (0)	0 (0)
Dropouts, *n* (%)	0 (0)	0 (0)	0 (0)

## Data Availability

The data presented in this study are available on request from the corresponding author.
